# The Comprehensive Healthcare Integration Framework: Integrating Behavioral Health Into Geriatric Primary Care

**DOI:** 10.1093/geroni/igaf122.654

**Published:** 2025-12-31

**Authors:** Anna Faul, Joseph D’Ambrosio

**Affiliations:** University of Louisville, Louisville, Kentucky, United States; University of Louisville, Louisville, Kentucky, United States

## Abstract

Integrating behavioral health into geriatric focused primary care involves collaboration between primary care and behavioral health clinicians to provide comprehensive, patient-centered care, addressing behavioral health needs alongside medical conditions. This model improves health outcomes, enhances behavioral health access, reduces stigma, and fosters holistic care. Emphasizing team-based collaboration, enables seamless coordination of treatment plans addressing physical and behavioral health needs, and is recognized as the gold standard for patient well-being. The Comprehensive Healthcare Integration Framework advances primary care behavioral health integration by outlining 8 evidence-based domains and 3 progressive constructs to guide primary care practices toward behavioral health integration. This integration requires person-centered strategies and interprofessional teamwork. At our Optimal Aging Clinic, a geriatric focused primary care practice, we developed and implemented integration workflows based on the CHI Framework to support comprehensive health care for older adults. Based on this model, we incorporate behavioral health consultants as generalists providing brief, focused interventions and biopsychosocial assessments to enhance primary care team capacity. We use a collaborative team of providers who all function at the top of their licenses. Our model ensures efficient, evidence-based management of complex cases and unmet clinical goals addressing physical, psychological, and social needs, improving care coordination and outcomes. Evaluation data shows improved management of depression and anxiety among older adults, together with overall enhanced biopsychosocial health, fostering better healthcare engagement from older adults in their care. This session will highlight our successes, the replicability of our model, and the challenges faced in integrating behavioral health into age-friendly care.

